# Functional connectivity underlying hedonic response to food in female adolescents with atypical AN: the role of somatosensory and salience networks

**DOI:** 10.1038/s41398-019-0617-0

**Published:** 2019-11-07

**Authors:** Gaia Olivo, Christina Zhukovsky, Helena Salonen-Ros, Elna-Marie Larsson, Samantha Brooks, Helgi B. Schiöth

**Affiliations:** 1 Department of Neuroscience, Functional Pharmacology, Uppsala University, Uppsala, Sweden; 2 Department of Neuroscience, Child and Adolescent Psychiatry, Uppsala University, Uppsala, Sweden; 3 Department of Surgical Sciences, Radiology, Uppsala University, Uppsala, Sweden; 4 Department of Human Biology, University of Cape Town, Cape Town, South Africa; 5 School of Natural Sciences and Psychology, Research Centre for Brain & Behaviour, Byrom Street, Liverpool, UK; 6 Institute for Translational Medicine and Biotechnology, Sechenov First Moscow State Medical University, Moscow, Russia

**Keywords:** Psychology, Neuroscience, Pathogenesis, Psychiatric disorders

## Abstract

Atypical anorexia nervosa (AN) usually occurs during adolescence. Patients are often in the normal-weight range at diagnosis; however, they often present with signs of medical complications and severe restraint over eating, body dissatisfaction, and low self-esteem. We investigated functional circuitry underlying the hedonic response in 28 female adolescent patients diagnosed with atypical AN and 33 healthy controls. Participants were shown images of food with high (HC) or low (LC) caloric content in alternating blocks during functional MRI. The HC > LC contrast was calculated. Based on the previous literature on full-threshold AN, we hypothesized that patients would exhibit increased connectivity in areas involved in sensory processing and bottom-up responses, coupled to increased connectivity from areas related to top-down inhibitory control, compared with controls. Patients showed increased connectivity in pathways related to multimodal somatosensory processing and memory retrieval. The connectivity was on the other hand decreased in patients in salience and attentional networks, and in a wide cerebello-occipital network. Our study was the first investigation of food-related neural response in atypical AN. Our findings support higher somatosensory processing in patients in response to HC food images compared with controls, however HC food was less efficient than LC food in engaging patients’ bottom-up salient responses, and was not associated with connectivity increases in inhibitory control regions. These findings suggest that the psychopathological mechanisms underlying food restriction in atypical AN differ from full-threshold AN. Elucidating the mechanisms underlying the development and maintenance of eating behavior in atypical AN might help designing specific treatment strategies.

## Introduction

Atypical anorexia nervosa (AN) is a restrictive eating disorder (ED) sharing the same diagnostic criteria of AN, except for the severely low weight^1^. In fact, patients with atypical AN are typically overweight or even obese before the onset of disorder^2,3^, usually occurring during adolescence^2,3^. As a result, they are in the normal-weight range when they come to psychiatric observation, despite often presenting with signs of medical instability^2,3^.

Neuroimaging research could help shedding light on how the brain circuitry can contribute to the development and maintenance of EDs^4^. In particular, functional neuroimaging studies in full-threshold AN have led to identify hyperactivity in bottom-up and top-down circuitry as underlying restrictive food choices^5^. Increased activity in the mesolimbic reward pathways has been consistently reported in patients with full-threshold AN, particularly in adults in response to monetary tasks or food-related visual or gustatory cues^5^. Few studies performed on adolescents confirmed a hyperactive mesolimbic response to monetary reward^5^. The top-down frontal circuitry is also hyperactive in full-threshold AN, leading to cognitive restraint over eating. In particular, top-down control mechanisms have been proposed to underlie the persistence of maladaptive behavior and cognitive inflexibility in these patients^5^.

Research on adolescents is nonetheless scarce. This is an important drawback, as adolescents are most at risk for developing EDs^2,3^. Moreover, brain plasticity is remarkably dynamic during adolescence^6^. Therefore, an early intervention targeting specific social and cognitive processes might be of particular importance at this stage, for preventing the chronicization of the disorder. In addition, no research has been undertaken on adolescents with atypical AN. Patients with atypical AN are often regarded as less severely affected than patients with full-threshold AN, but that is actually not the case, as they present the same prevalence of medical complications^2,3^. Moreover, they tend to exhibit higher restraint over eating and body dissatisfaction, as well as lower self-esteem, compared to patients with full-threshold AN^3^.

In previous work in our cohort of atypical AN patients, we have reported a reduction in resting-state functional connectivity in areas involved in social cues and face processing^7^, in the context of preserved gray matter volume^8^ and white matter microstructure^9^. In the current exploratory work, we have investigated functional circuitry underlying hedonic responses to food in 28 female adolescent patients diagnosed with atypical AN and 33 healthy controls. Hedonic responses to food are one of the main factors leading personal food choices and food intake^10–12^. A recent review^13^ has outlined the main neural alterations observed in adults with full-threshold AN in response to active viewing of food pictures; in particular, participants were required to either imagine eating the food shown, or to rate food cues according to their pleasantness^13^. Full-threshold AN patients showed lower activity compared with controls during visual processing of food in the inferior parietal lobule and lateral prefrontal cortex^14,15^; this was hypothesized to be possibly reflective of food-induced weight and body shape concerns^16–18^. Moreover, the simultaneous hyperactivation of medial prefrontal regions was suggested to indicate top-down control efforts aimed at restraining eating and food avoidance^16,17,19,20^. In addition, lower activation in the medial orbitofrontal cortex and insula during visual processing of food cues has been linked to lower hedonic reactivity in patients with AN compared with controls^14,18^. However, a recent study on adolescents with full-threshold AN showed higher bottom-up, as well as top-down activity in patients in response to high calories food compared with controls^21^. In particular, contrary to what has been reported in adults, high calories food images elicited increased activity in the insula, involved in somatosensory integration and reward attribution, coupled to higher activity in the inferior frontal gyrus (IFG), involved in salience bottom-up responses, and medial prefrontal cortex, involved in top-down control and reappraisal strategies. Based on findings in adolescents, we hypothesized that our patients would exhibit: (a) increased connectivity in areas involved in somatosensory integration, such as the insula^21^ and the thalamus;^22,23^ (b) higher connectivity in areas involved in salient bottom-up responses, such as the medial prefrontal cortex, identified in patients with full-threshold AN, and in the orbitofrontal cortex, identified in overweight adolescents^24^ and dieting individuals;^25^ (c) a compensatory increase in connectivity from areas related to top-down inhibitory control compared with controls, particularly in the medial prefrontal cortex and in the dorsolateral prefrontal cortex (DLPFC). Indeed, the DLPFC has been reported to be more active in adolescents on a successful diet, compared with overweight and lean controls, in response to food images^26^. Moreover, the DLPFC is more active in obese children compared with controls when viewing food pictures, with an inverse correlation with self-esteem;^27^ as atypical AN patients often start out as overweight/obese and subsequently successfully restrain their caloric intake in order to lose weight, we expected higher DLPFC activity in our patients compared with controls when viewing caloric food pictures.

## Methods

### Participants

The protocol was approved by the regional ethics committee (Etikprövningsnämnden) of Uppsala, Sweden, and complied with the ethical standards of the relevant national and institutional committees on human experimentation and with the Helsinki Declaration of 1975, as revised in 2008. All participants and their guardians gave written consent to participate in the study.

Thirty-one adolescent female outpatients (mean age 14.8 years) were recruited by the Eating Disorder Unit (EDU) of the Department of Child and Adolescent Psychiatry at the Uppsala University Hospital, Uppsala, Sweden. All patients received a diagnosis of restrictive atypical AN according to the fifth edition of the DSM-5^1^, as they presented with features of AN but were above −2 body mass index (BMI) standard deviations (SDS) for age^28^. BMI standard deviations (BMI-SDS) per age were calculated, according to the World Health Organization reports (https://www.who.int/growthref/who2007_bmi_for_age/en/). Menstrual status was also recorded. A pediatrician with experience of ED and associated with an ED clinic was responsible for the initial assessment of the patients, consisting of a structured protocol including: the history of the ED, medical history (including medical and psychiatric comorbidities), menstrual status, demographics, and physical examination (including weight and height measurements). The patients were then referred to a psychiatrist at the EDU, who confirmed the diagnoses and further assessed the patients with the diagnostic instruments included in the “Stepwise” data collection system^29^. The “Stepwise” data collection system was introduced in Sweden since 2014 and is used by all specialized ED services. It comprises semi-structured diagnostic interviews, clinical ratings and self-ratings, automated follow-up schedules and administrative functions^29^. The MINI-KID interview^30^ was used to further screen for comorbid diseases. Furthermore, the history of weight and height changes was obtained from the growth charts provided by the school health services. Treatment was started after the diagnosis, and consisted of family-based intervention, aiming at helping the parents to take a leading role against the ED. Parents were advised on regular meals and meal sizes; however, a standardized diet was not provided, to comply with the specific family necessities and situation. Patients had regular visit at the EDU; at each visit, their weight progress was assessed by a nurse, and the ED status were assessed by the psychiatrist. Forty female healthy controls (mean age 15.3 years) were recruited from local schools through advertisement. Inclusion criteria for all participants were age between 13 and 18 years and right-handedness. Patients and controls were excluded from the study if they had past or current history of psychiatric disorders (apart from current ED in patients), past or current pregnancy, comorbid neurological diseases, metallic implants, or claustrophobia. They were also excluded if they made use of psychotropic medication or if they had history of alcohol, drug, or medication abuse. Approximately 50% of new patients were excluded according to these criteria. Controls were further excluded if they had a BMI < −2 BMI-SDS and an EDE-Q total score > 2.0, which has been suggested as the optimal cut-off to distinguish between the clinical and the general population^31^.

ED-related cognition was assessed via a 38-items self-reported questionnaire, the EDE-Q^32^, youth version. The EDE-Q comprises four subscales measuring specific features of the ED behavior: Restraint, Eating Concern, Shape Concern, Weight Concern. Depression symptoms were assessed with the MADRS-S^33^, self-reported.

### MRI acquisition and protocol

The participants were instructed to eat before coming to the hospital. Prior to the scanning, they were asked to rate their satiety on a visual scale ranging from 1 to 10. If they indicated a score below 3, they were provided with something to eat, like cookies or snacks. The scanning procedure was carried out within 60 days of the initial visit at the clinic. The diagnosis of atypical AN was still confirmed by the psychiatrist at the time of scanning. Structural and functional brain images were acquired with a Philips 3-T scanner (Achieva, Philips Healthcare, Best, Netherlands) using a standard head coil. Structural images were acquired with a T1-weighted turbo-field-echo (TFE) sequence (TR = 8100 ms; TE = 3.7 ms; flip angle: 8°; slice thickness = 1 mm; slice spacing = 1 mm). 125 volumes were registered during the T2*-weighted echo-planar imaging sequence (TR = 3000 ms; TE = 35 ms; flip angle = 90 °; slice thickness = 3 mm; slice spacing = 1 mm gap; in-plane resolution: (3 mm × 3 mm).

For the fMRI acquisition, images of food were presented in the scanner using MRI-compatible goggles (NordicNeuroLab, Bergen, Norway) attached to the headcoil. Participants were shown images of low calorie (LC) food, high-calorie (HC) food, or a gray screen with crosshair in the center (X) in five cycles of alternating blocks with the following pattern: X, LC, X, HC. Each block contained six images, shown at 3 s intervals. No images were repeated. 54 measurements were acquired for X, 30 for LC and 30 for HC. Participants were instructed to imagine what it felt like to eat the food presented. Food images were selected based on their familiarity according to local palate and on their caloric content as perceived by a focus groups representative of the population to be studied. Food images were controlled for visual features (color, size, etc.). A description of our fMRI paradigm can also be found in Wiemerslage et al.^34^.

### MRI pre-processing

Initial pre-processing was carried out with Statistical Parametric Mapping (SPM) 12 (https://www.fil.ion.ucl.ac.uk/spm/), running on a Matlab 2017a platform. Slice-timing correction was performed on functional images, followed by realignment to correct for head motion. One patient and four controls had moved more than 3 mm; they were thus excluded from further analyses. Functional images were coregistered to structural images. The “old segmentation” was applied to structural images to generate GM, WM and cerebrospinal fluid probability maps using the adolescent tissue probability maps provided by the Imaging Research Center at Cincinnati Children’s Hospital Medical Center (CCHMC; https://irc.cchmc.org/software/pedbrain.php), version 2 (9/2002). In order to better accommodate our adolescent sample, the “old” sample template was used (age range: 13–18 years). Structural and functional images were normalized to Montreal Neurological Institute (MNI) standard brain template^35^ using the deformation parameters derived from the segmentation procedure, with 3 mm^3^ voxel size. Results from the normalization procedure were visually inspected, and two patients and two controls were excluded from further analyses due to normalization imperfections. Twenty-eight patients and thirty-three controls were retained. Functional images were smoothed with a four full width at half maximum (FWHM) Gaussian kernel to increase the signal-to-noise ratio and to accommodate for anatomical and functional variability between subjects.

Pre-processed structural and functional images were imported in CONN connectivity toolbox (https://www.nitrc.org/projects/conn). Band-pass filtering was applied, to retain data in the 0.01–0.1 Hz band)^36–38^. and reduce the interference of physiological noise occurring at higher frequencies^39,40^. Linear detrending was applied, and the effects of white matter, motion and cerebrospinal fluid were regressed out. First level analysis was performed by including motion parameters as covariates.

### Statistical analysis of clinical data

Statistical analyses of clinical measures were performed with Statistical Package for Social Science (SPSS) v. 24 (https://www.ibm.com/analytics/spss-statistics-software). Four separate ANOVA tests were carried out to test for group differences on age, BMI, BMI-SDS per age, EDE-Q and MADRS-S total scores. The threshold for significance was set at *p* < 0.05. BMI-SDS loss in patients was calculated as the difference between the BMI-SDS per age from the moment of maximum documented weight until the moment the diagnosis of atypical AN was made (Table 1).

**Table 1:** Sample description

	Patients (mean ± SD)	Controls (mean ± SD)	Sig.
Age (years)	14.8 ± 1.6	15.3 ± 1.3	<0.184
BMI (Kg/m^2^)	19.4 ± 2.4	20.9 ± 2.3	<0.014^a^
BMI-SDS	−0.36 ± 0.85	0.11 ± 0.71	<0.022^a^
EDE-Q	3.5 ± 1.7	0.6 ± 0.8	<0.001^a^
MADRS-S	22.5 ± 11.6	5.2 ± 5.4	<0.001^a^
Disease duration (years)	0.72 ± 0.45	–	–
BMI-SDS loss^b^	1.00 ± 0.72	–	–
Diagnosis-to-scan (days)	30.9 ± 12		
BMI gain prior to scanning (Kg/m^2^)^c^	0.79 ± 0.63	–	–

^a^Significant with *p* < 0.05

^b^From the moment of maximum documented weight to diagnosis

^c^From clinical assessment to scanning

### Functional connectivity analysis

A seed-to-voxel analysis was performed with CONN toolbox. CONN provides a complete brain parcellation including 91 cortical areas and 15 subcortical areas from the FSL Harvard-Oxford Atlas, and 26 cerebellar areas from the AAL atlas, for a total of 132 seeds. Functional connectivity maps from each of these seeds were generated, and tested for differences between patients and controls. Age and BMI-SDS were entered as covariates in the analysis. The primary voxel-level threshold was set at *p* < 0.001^41^. A further correction for Family-wise error rate (FWE) at cluster level was applied to voxels surviving the primary threshold^41^. The final threshold was then set at *p* < 0.0004 (0.05/132), FWE-corrected, to account for the number of seeds tested.

Moreover, separate regression analyses were carried out separately in patients and controls, to test for correlations of BMI, EDE-Q and MADRS-S scores on connectivity in clusters found to be significantly different between groups. To this purpose, correlation coefficients were extracted and imported in SPSS. Within patients, functional connectivity was also tested for correlations with disease duration and weight loss from the moment of maximum documented weight to the diagnosis, expressed as BMI-SDS loss. All models were corrected for age. The same above-mentioned thresholding was applied. As nine cluster of altered connectivity were identified at the main analysis, the Bonferroni correction for multiple testing led to a final threshold of *p* < 0.002 in controls (9 clusters * 3 clinical measures) and *p* < 0.001 in patients (9 clusters * 5 clinical measures).

## Results

### Clinical measures

Patients had a mean disease duration of 8.6 months, and their BMI-SDS per age had reduced by one unit since the moment of maximum documented weight. Average treatment duration to the time of scanning was 1 month, with an average BMI gain of 0.8 Kg/m^2^. Nine of our patients had secondary amenorrhea, two were on oral contraceptives, three were in premenarche, and 14 had menstruation. Patients and controls did not differ on age (*p* < 0.184); however, they were significantly different on BMI (*p* < 0.014), BMI-SDS per age (*p* < 0.022), and on the EDE-Q (*p* < 0.001) and MADRS-S total scores (*p* < 0.001) (Table 1).

### Functional connectivity analysis

**Fig. 1:**
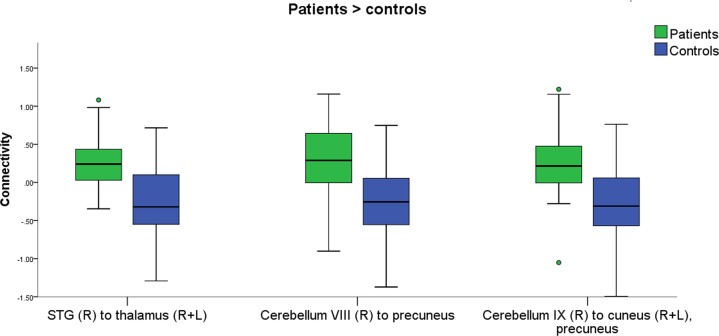
Increased connectivity in patients compared with controls. The boxplot shows the clusters were increased connectivity was found in patients (green) compared with controls (blue), in response to food with high vs. low caloric content. Circles represent cases lying between 1.5 and 3 times the interquartile range (mild outlier). The results were verified in SPSS by removing the outliers (see methods). Connectivity values were extracted with SPM. L left, R right, STG superior temporal gyrus

**Fig. 2:**
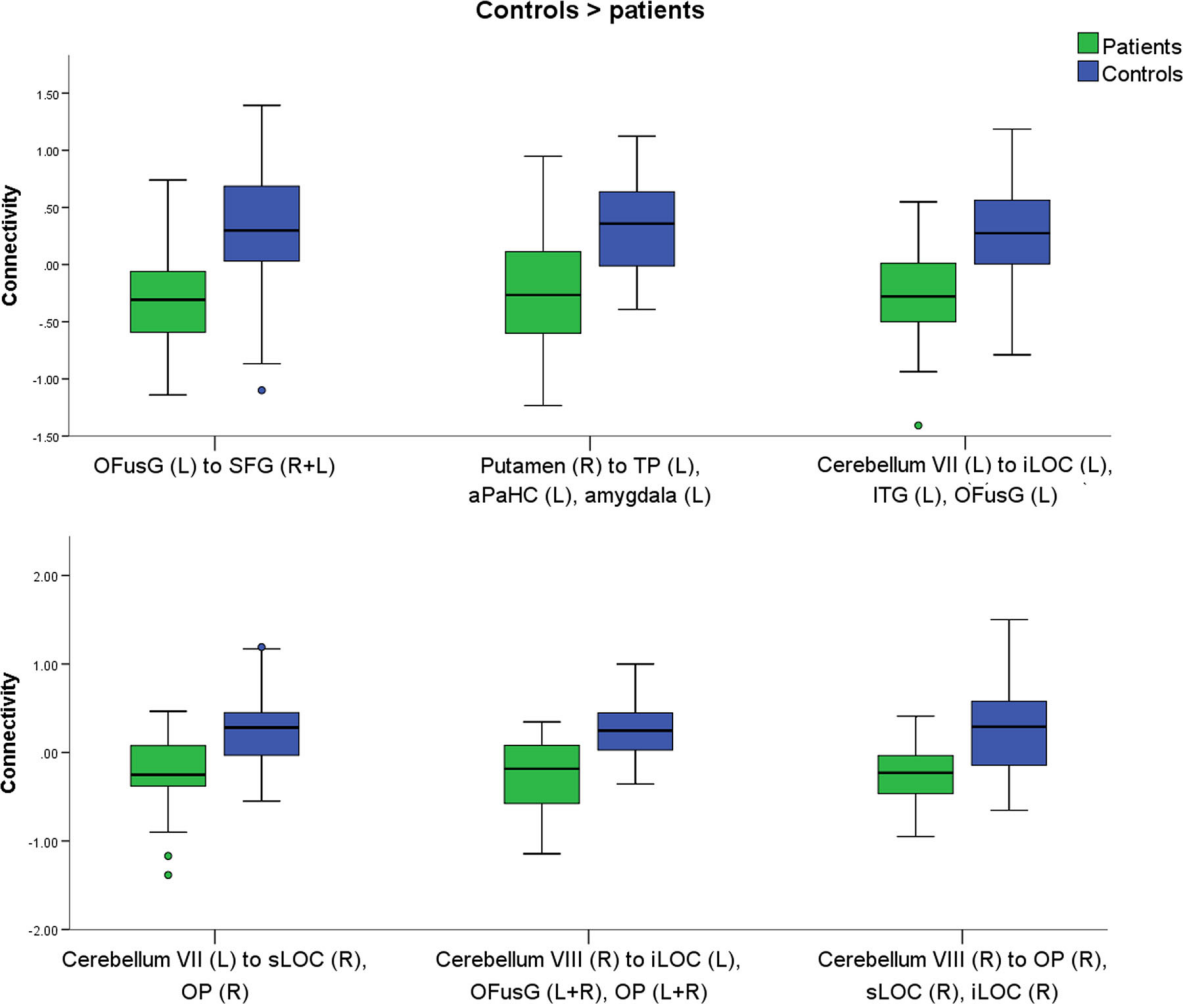
Reduced connectivity in patients compared with controls. The boxplot shows the clusters were reduced connectivity was found in patients (green) compared with controls (blue), in response to food with high vs. low caloric content. Circles represent cases lying between 1.5 and 3 times the interquartile range (mild outlier). The results were verified in SPSS by removing the outliers (see methods). Connectivity values were extracted with SPM. aPaHC anterior parahippocampal cortex, iLOC = inferior lateral occipital cortex, ITG inferior temporal gyrus, L left, OFusG occipital fusiform gyrus, OP occipital pole, R right, SFG superior frontal gyrus, sLOC superior lateral occipital cortex, TP temporal pole

**Fig. 3:**
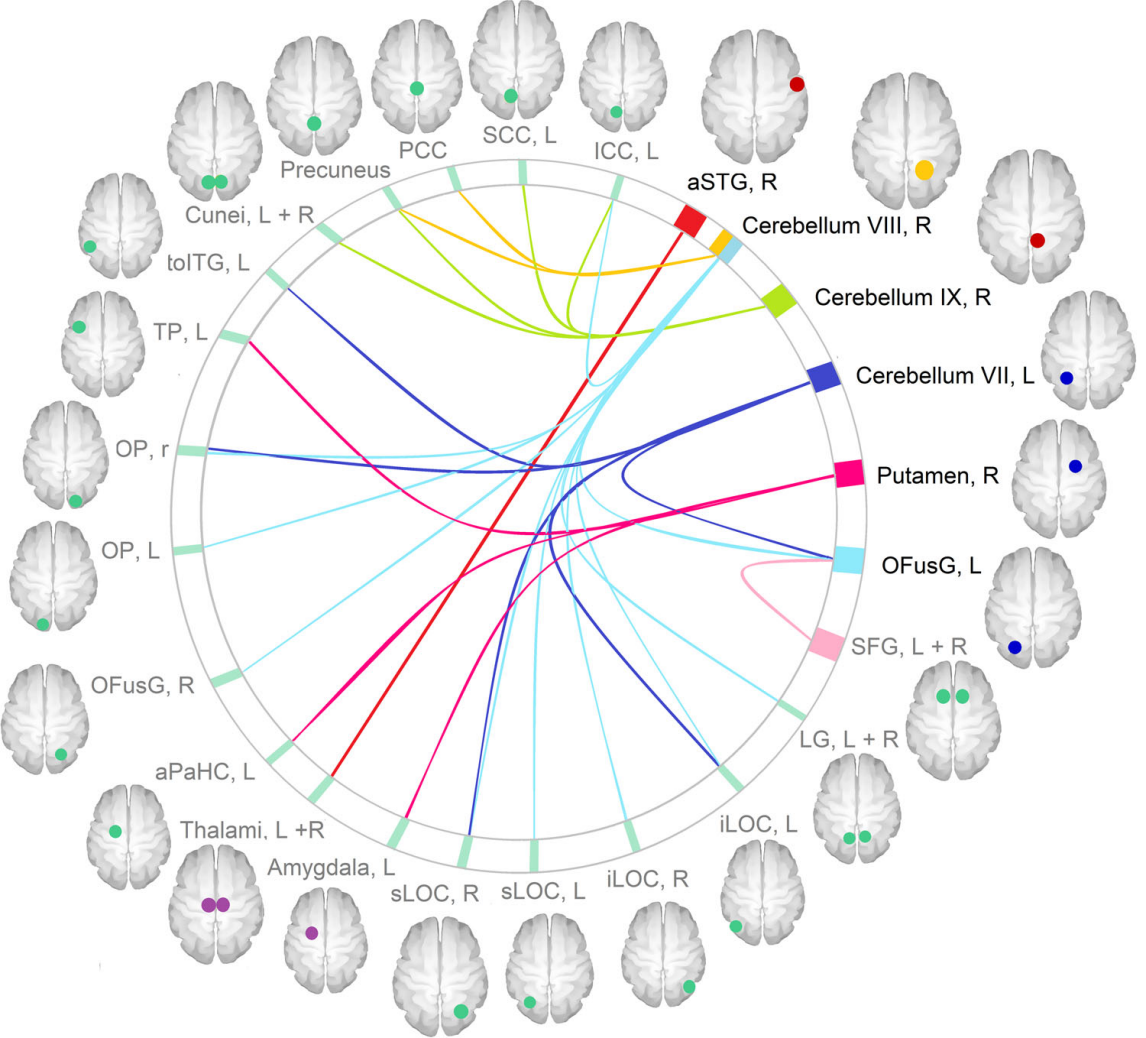
Functional connectivity in patients vs controls. The figure summarizes group differences in seed-to-target functional connectivity when viewing picture of high calories vs low calories food. The seeds with greater connectivity in patients compared with controls are represented in red; the seeds with lower connectivity in patients are represented in blue; the target regions are represented in green. The cerebellar lobule VIII, R is represented in orange as it showed both increased and reduced connectivity to different target regions. The image was created with CONN toolbox. The target regions are depicted as circles merely for representative purposes. The exact peak coordinates and voxel extent of each target cluster are reported in Table 2. aPaHC anterior parahippocampal cortex, aSTG anterior superior temporal gyrus, ICC intracalcarine cortex, iLOC inferior lateral occipital cortex, L left, LG lingual gyri, OFusG occipital fusiform gyrus, OP occipital pole, PCC posterior cingular cortex, R right, SCC supracalcarine cortex, SFG superior frontal gyri, sLOC superior lateral occipital cortex, toITG temporo-occipital inferior temporal gyrus, TP temporal pole

Patients exhibited patterns of increased and reduced connectivity compared with controls. When viewing HC vs. LC food images, connectivity was higher in patients from the right anterior superior temporal gyrus (aSTG) to the thalamus (*p* < 0.0003, FWE-corrected), from the right cerebellar lobule VIII to the precuneus and posterior cingulate cortex (PCC) (*p* < 0.00006, FWE-corrected), and from the right cerebellar lobule IX to the cuneus bilaterally, to the precuneus, and to the left intracalcarine cortex (ICC) and supra-calcarine cortex (SCC) (*p* < 0.00002, FWE-corrected) (Fig. 1). On the other hand, connectivity was lower in patients compared with controls from the left occipital fusiform gyrus (OFusG) to the superior frontal gyri (SFG) bilaterally (*p* < 0.0002), from the right putamen to the left temporal pole (TP), anterior parahippocampal cortex (aPaHC) and amygdala (*p* < 0.0002 FWE-corrected), from the left cerebellar lobule VII to the left OFus G, temporo-occipital inferior temporal gyrus (ITG) and occipital areas (*p* < 0.00003, FWE-corrected), and from the right cerebellar lobule VIII to several occipital and cerebellar areas (*p* < 0.000001, FWE-corrected) (Fig. 2), when viewing HC vs. LC food images. Results from the connectivity analyses reported in Table 2, and summarized in Fig. 3. No correlations were found between connectivity in the affected functional connections and clinical measures.

**Table 2:** Functional connectivity differences between patients and controls

Seed	Target	MNI coordinates			C_E_	*P* FWE-corr
		*x*	*y*	*z*		
*Patients* *>* *controls*						
aSTG (R)	Thalamus (R + L)	10	−26	20	207	<0.0003
Cerebellum (VIII, R)	Precuneus, PCC	6	−58	34	253	<0.00006
Cerebellum (IX, R)	Cuneus (R + L) Precuneus ICC (L) SCC (L)	−2	−68	16	291	<0.00002
*Patients* *<* *controls*						
OFusG (L)	SFG (R + L)	4	56	34	203	<0.0002
Putamen (R)	TP (L) aPaHC (L) Amygdala (L)	−32	10	−28	216	<0.0002
Cerebellum (VII, L)	iLOC (L) toITG (L) OFusG (L)	−48	−64	−8	404	<0.000001
Cerebellum (VII, L)	sLOC (R) OP (R)	28	−82	34	273	<0.00003
Cerebellum (VIII, R)	iLOC (L) OFusG (L + R) OP (L + R) sLOC (L) ICC (L) LG (L + R) Cerebellum	−26	−70	−14	2558	<0.000001
Cerebellum (VIII, R)	OP (R) sLOC (R) iLOC (R)	28	−86	28	570	<0.000001

### Mild outliers

When inspecting the box plots (Fig. 1, Fig. 2), few individuals were lying between 1.5 and 3 times the interquartile range (mild outliers). When no specific reasons for outliers are detected (e.g. MRI artefacts, excessive movement, normalization issues), mild outliers are likely to represent normal variability in the data at population level. We had inspected the raw and pre-processed data to exclude the presence of artefacts and ensure the good quality of the normalization. Cases with excessive movements were discarded during the first steps of pre-processing. Thus, no instrumental reasons for mild outliers were noticed. Nonetheless, we performed a series of ANCOVAs corrected for age and BMI-SDS in SPSS after the removal of the outliers, to ensure that our results were not driven by mild outliers. All results were confirmed.

## Discussion

We investigated neural connectivity underlying the hedonic response to food images in a sample of 28 adolescent females diagnosed with atypical AN, and 33 age- and sex-matched healthy controls. Different connections showed higher or lower functional connectivity in patients compared with controls, when watching images of food with high vs. low caloric content.

### Higher connectivity in patients in somatosensory areas

Patients showed higher connectivity from the right cerebellar lobule IX to the cuneal cortices bilaterally, to the precuneus, extending to the primary visual cortex and SCC, in response to food with high vs. low caloric content. These areas are all intercalated on the visual stream. Additionally, the precuneus is involved in visuo-spatial imagery and in episodic memory retrieval^42^. It has also been related to appetite control, as its activity is elicited when asking subjects to exert cognitive inhibitory control over the urge to eat the food shown in a picture^43^. Moreover, both the cuneus and precuneus have been related to early imagery processing^44^. The cerebellar lobule IX is also part of the default-mode network^45^, and receives sensory inputs from the trigeminal nerve^46^, which provides motor innervation to the masticatory muscles and tactile and nociceptive innervation to the face and mouth mucosae. It receives also tactile inputs from the hands^45^. The higher connectivity within this network might underlie an enhanced capacity for visual and tactile imagery of the food shown, in line with our hypothesis of increased somatosensory processing in patients.

Moreover, the connectivity from the right aSTG to the thalamus, bilaterally, was greater in patients compared with controls when viewing (and imaging to eat) HC vs. LC foods. The STG’s main function has been traditionally related to the auditory pathway, however its function seems to be more complex, and more broadly related to the multimodal integration of sensorial stimuli through its rich connections with the thalamus^47,48^. Indeed, the thalamus acts as a sensory relay structure and as a hub on sensory pathways^23^. Interestingly, the thalamus is strongly involved in gustatory processing^49,50^ and food intake regulation^22^, while the STG has been previously found to be more active in response to the presentation of food cues compared with non-food matched stimuli of different modalities^43,51,52^. In particular, the STG was more responsive to high calories food irrespective of the drive to eat^53^. Thus, when imaging eating a food with higher caloric content, the connectivity between associative and gustatory areas is actually increased in patients compared with controls, supporting our hypothesis of an increased somatosensory elaboration of food stimuli.

Finally, the connectivity from the right cerebellar lobule VIII to the precuneus, extending to the PCC, was also higher. The cerebellar lobule VIII is part of the sensorimotor cerebellum, active during sensorimotor and somatosensory tasks^45,54^. The activity of the PCC in response to food images is modulated by the caloric content of the food displayed^55^. Interestingly, the PCC has been largely implicated in self-referential processing during mind-wandering or judgemental tasks, and it has been proposed to be specifically involved in cognitive processing of self-reference^56^, defined by Brewer et al.^56^. as “getting caught up in” one’s experience, such as in drug craving or in a particular viewpoint^56^. The precuneus, on the other hand, is involved in self-centered thinking and episodic memory retrieval^42^. The higher connectivity between these regions might therefore support an enhanced brain response to the imaginary eating of food with higher caloric content. However, this interpretation has to be considered speculative, and more research has to be undertaken to specifically investigate food-related self-referential processing in these patients.

### Lower connectivity in patients in areas related to salience attribution and inhibitory control

The connectivity was lower in patients compared with controls when watching images of food with high, compared to low, caloric content, from the left OFusG to the SFG bilaterally. The fusiform gyrus is involved in semantic memory^57,58^. Meta-analyses, however, have also demonstrated the involvement of the fusiform gyrus in visual processing of food images in both adolescents and adults^52,59^. Interestingly, connectivity of the fusiform gyrus has been reported to be decreased in AN patients^60^. The SFG, on the other hand, is involved in introspection and in self-related processing^61^, as well as in exerting inhibitory control over appetite in response to visual food cues^43^. This finding seems to be in contrast with our hypothesis of an increase top-down inhibitory control over appetite and food-related visual stimulation. However, this might be due to an overall reduced rewarding value for the caloric content of food. In fact, patients also showed reduced connectivity from the right putamen to the left TP, aPaHC and amygdala in response to high calories compared with low calories food. These areas are part of the reward system^62^ and salience network^63^. The putamen has been reported to be more active in response to low calories rather than high calories food in satiated females^64^. Moreover, the amygdala-striatal circuitry underlies impulsive behaviors^64^, and activity in the amygdala and in the right para-hippocampal gyrus are elicited by food cues in obese individuals^64^. Thus, in our atypical AN patients, imaging to eat the HC foods shown is associated to a weaker connectivity in the salience network compared to controls, and this might explain the subsequent lack of recruitment of inhibitory control regions in response to visual processing of food cues.

The connectivity was also lower in patients compared with controls from the left cerebellar lobule VII to the left OFus G, left temporo-occipital ITG and left occipital areas. The cerebellar lobule VII is part of the posterior cerebellum, also known as the cognitive cerebellum^45^. Activity in this lobule is elicited by executive, working memory and affective tasks^45^. In particular, the cerebellar lobule VII seems to be consistently active when viewing emotional vs. neutral stimuli^45,54^. The OFusG and the ITG are part of the ventral visual stream, involved in objects recognition^65^. As mentioned previously, the fusiform gyrus is also involved in visual processing of food images^52,59^, and of meaningful stimuli in general^66^. Indeed, the left visual stream in particular appears to be more involved in the analytic, attention-dependent processing of objects^65^. The LOC, on the other hand, beside its role in object recognition, is also important in tactile perception, serving as a hub for recognition of tactile imagery^67^. Interestingly, LOC activation seems to be independent of object familiarity^68^. Thus, the lower connectivity between the cerebellar lobule VII and the left ventral visual stream is in line with a failure in engaging patients’ attention with foods with high vs. low caloric content.

Finally, the connectivity was lower in patients when viewing high calories food images from the right cerebellar lobule VIII to several occipital and cerebellar areas. As mentioned above, the cerebellar lobule VIII is part of the sensorimotor cerebellum, active during sensorimotor and somatosensory tasks^45,54^. The cerebellar lobule VIII is particularly engaged during tactile tasks, as it stores a representation of the hand^54^. Most of the target areas with decreased connectivity are primarily involved in visual processing, although recently some of them have been also involved in association processing^67^. The LOC in particular is a hub for multimodal integration of visual stimuli, and is particularly involved in tactile imagery and in decoding of objects’ shapes, forms, and orientations in a multimodal perspective^67^. The finding of a reduced connectivity between these areas is of difficult interpretation, and will need to be further explored by future studies specifically targeting the cerebellum and involving different visual stimuli.

### Implications for treatment

In full-threshold AN, the aberrant eating behavior is likely to be facilitated by increased cognitive control and reappraisal strategies toward salient food stimuli^21^, and sustained by lack of cognitive flexibility^69,70^, leading to difficulties in changing the adopted behavioral patterns despite adverse consequences. In our patients, however, the neurobiological mechanisms underlying food restriction seems to differ from full-threshold AN, as HC food images were not associated to increased bottom-up response nor to increased top-down response. The specific neuro-behavioral mechanisms underpinning restraint over eating in atypical AN needs further investigation, as it can have relevant implications for treatment. Indeed, though Family-based treatment (FBT) is currently the leading therapy for adolescents with full-threshold AN^71^, the risk for relapse, particularly in the first 16 months after treatment, is nonetheless still high^72–74^. Enhanced cognitive behavioral therapy (CBT-E), targeting the specific psychopathological mechanisms sustaining the ED in each individual, seems to be a promising alternative treatment. Thus, elucidating the mechanisms underlying the development and maintenance of unhealthy eating habits in atypical AN might help designing and improving specific treatment strategies. However, it has to be noted that none of the clinical scores tested were significantly associated with the functional connectivity coefficients in the detected seed-to-target pairs, as would have been expected if they contributed to within-group variability in symptoms severity. Thus, their potential prognostic value needs further investigation.

### Limitations

Our study has some limitations according to the recent guidelines for neuroimaging studies in EDs suggested by^4^, and has thus to be considered of exploratory nature; nonetheless, it laid the foundations for future research on atypical AN. The sample size was adequate for fMRI studies^75^; however, future studies on larger sample will be needed to verify our findings, and ideally complemented with food ratings by the participants. Moreover, there was a delay of up to 60 days between the diagnosis and the scanning procedure, and patients had undergone treatment during that period. This delay was due to the informed consent protocol and hospital procedures. In fact, patients and their guardians were provided with information regarding the study, and given one week to decide whether they wanted to participate. Then, the MRI scan had to be booked according to the patients’ and hospital necessities, leading to a certain delay. Patients did, however, start the treatment right away, due to ethical reasons. It must be noticed that all patients were still diagnosed with atypical AN at the time of scanning; however, whether early-onset effects of the treatment might have affected our results needs to be clarified. The meal schedule was not standardized between patients; however, all patients were satiated before scanning. We did not have information pertaining the menstrual status of the controls, thus we could not control our findings for menstrual status. Moreover, though the treatment protocol required patients not to exercise, non-compliance cannot be ruled out. Finally, the controls did not undergo a complete psychological examination, and their mental health history was self-reported. However, using the subclinical cut-off on the EDE-Q as exclusion criterion ensured the exclusion of controls with possible underdiagnosed EDs.

## Conclusions

We investigated neural connectivity underlying hedonic response to food images in a sample of 28 adolescent females diagnosed with atypical AN, and 33 age- and sex-matched healthy controls. Based on previous literature on adolescents with full-threshold AN, we hypothesized that viewing food with high caloric content would be associated to increased connectivity in patients in areas involved in somatosensory integration and bottom-up responses, possibly coupled to a compensatory increase in connectivity from areas related to top-down inhibitory control, compared with controls. In accordance with our hypothesis, our results showed a stronger connectivity in patients in pathways related to the integration of multimodal somatosensory input and memory retrieval, in response to food with high vs. low caloric content. This, however, was coupled to a lower connectivity in salience and attentional networks, and reduced connectivity between areas involved in visual food cues processing and appetite regulatory regions, contrary to our hypothesis. Thus, food with high caloric content is associated to increased processing of somatosensory information in patients; however, it is attributed less salience and engage patients’ attention less than food with low caloric content. Consequently, no compensatory increases in inhibitory control regions are observed. Elucidating the mechanisms underlying the development and maintenance of unhealthy eating habits in atypical AN might help designing and improving specific treatment strategies. Thus, future studies will be needed to further elucidate the role of cerebellar-occipital connectivity and the role of visual areas in the pathogenesis of atypical AN, possibly using different visual stimuli or specifically targeting cerebellar circuitry, and complementing the fMRI protocol with food ratings by the participants.
